# Educational Attainment Better Protects African American Women than African American Men Against Depressive Symptoms and Psychological Distress

**DOI:** 10.3390/brainsci8100182

**Published:** 2018-09-30

**Authors:** Shervin Assari

**Affiliations:** 1 Department of Psychology, University of California, Los Angeles (UCLA), Los Angeles, CA 90095, USA; assarish@ucla.edu; Tel.: +1-(310)-206-5162; 2 BRITE Center for Science, Research and Policy, University of California, Los Angeles (UCLA), Los Angeles, CA 90095, USA; 3 Center for Research on Ethnicity, Culture, and Health (CRECH), School of Public Health, University of Michigan, Ann Arbor, MI 48104, USA; 4 Department of Psychiatry, University of Michigan, Ann Arbor, MI 48109, USA

**Keywords:** African Americans, socioeconomic status (SES), racism, distress, depression

## Abstract

Background: Recent research has shown smaller health effects of socioeconomic status (SES) indicators such as education attainment for African Americans as compared to whites. However, less is known about diminished returns based on gender within African Americans. Aim: To test whether among African American men are at a relative disadvantage compared to women in terms of having improved mental health as a result of their education attainment. This study thus explored gender differences in the association between education attainment and mental health, using a representative sample of American adults. Methods: The National Survey of American Life (NSAL; 2003) recruited 3570 African American adults (2299 females and 1271 males). The dependent variables were depressive symptoms and psychological distress. The independent variable was education attainment. Race was the focal moderator. Age, employment status, and marital status were covariates. Linear regressions were used for data analysis. Results: In the pooled sample that included both male and female African American adults, high education attainment was associated with lower depressive symptoms and psychological distress, net of covariates. Significant interactions were found between gender and education attainment with effects on depressive symptoms and psychological distress, suggesting stronger protective effects of high education attainment against depressive symptoms and psychological distress for female as compared to male African Americans. Conclusion: A smaller gain in mental health with respect to educational attainment for male African American males as compared to African American females is in line with studies showing high risk of depression in African American men of high-socioeconomic status. High-SES African American men need screening for depression and psychological distress.

## 1. Introduction

Socioeconomic status (SES) resources are associated with better mental health [1,2,3]. Education attainment, one of the most important SES indicators, shapes the social patterning of depressive symptoms and psychological distress [4]. It is unclear if the protective effect of education against depression and psychological distress depends on tertiary factors such as race and ethnicity.

According to the Minorities’ Diminished Return (MDR) theory [5,6], SES indicators show weaker effects on the physical and mental health of African American and other racial and ethnic minority groups as compared to whites. Education attainment and income have smaller protective effects with respect to depression [4], diet [7], smoking [8], alcohol use [9], impulse control [10], obesity [11,12], chronic disease [13,14], and mortality [15] for African Americans as compared to whites. In a 25-year follow up study, African American men with the highest education attainment were the only group who experienced an increase in depressive symptoms over time, a phenomenon which was not present for white men, white women, or African American women [4]. In a study of African American boys, high income was associated with an increased risk of major depressive disorder [2]. In another study, high-SES youth experienced more depressive symptoms over time [16,17]. In another study, family type protected white but not African American youth from anxiety [18]. In a study among Michigan residents, high income reduced risk of poor self-rated mental health for whites but not African Americans [19]. Finally, another study showed that high education was a risk factor for risk of suicidal ideation for black Caribbean females [20].

Although MDR theory [5,6] suggests that education attainment and other SES indicators have a smaller protective effect on African Americans than whites, it is unclear why this pattern is more pronounced for males than females [2,3,4]. To fill this knowledge gap in the literature, the current study explored gender differences in the protective effects of education attainment on depressive symptoms and psychological distress in a nationally representative sample of African American adults in the United States.

## 2. Methods

### 2.1. Design

The National Survey of American Life (NSAL) is a cross-sectional mental health survey of non-Hispanic African American adults in the United States [21]. Although a full and detailed description of NSAL methods and sampling is available elsewhere [21], we briefly report the study design and methodology here.

### 2.2. Participants and Sampling

The NSAL applied a household probability sampling to draw a national sample of adults. The NSAL African Americans were selected from rural areas, large cities, and other urban areas [22,23,24]. With a multi-stage sampling design, the NSAL used a core national sample of African Americans and whites which was almost identical to the National Survey of Black Americans (NSBA). The NSAL participants were adults (aged 18 years and older) who resided in the coterminous United States (48 states). Participants were restricted to non-institutionalized individuals who could conduct a structured interview in English. As a result, individuals were excluded if they resided in nursing homes, long-term medical care settings, prisons, and jails [21]. The analytical sample of this study included a total number of 3570 African American adults (2299 females and 1271 males).

### 2.3. Data Collection

Structured interviews were used to collect data. Interviews were conducted in English. About 82% of total interviews were face-to-face, while the 18% remaining were conducted as telephone interviews. The NSAL used a computer-assisted personal interview (CAPI) for all the face-to-face interviews. The CAPI uses computers to assist the process of answering lengthy questionnaire with multiple skip patterns. CAPI enhances data quality, at least for long and complex surveys. Interviews took about 140 min to complete. Response rate was 71% for African Americans.

### 2.4. Measures

The variables employed for this study were race/ethnicity, age, household income, education attainment, and perceived (every day) discrimination.

Race/Ethnicity: NSAL measured race/ethnicity as self-identified race and ethnicity. Participants self-identified either as non-Hispanic African Americans or non-Hispanic whites. Non-Hispanic African Americans were defined as black without any ancestral ties to Caribbean countries.

Income: Household income was measured using self-reported data. Household income was measured as: (1) US$0–9999; (2) US$10,000–19,999; (3) US$20,000–39,999; and (4) US$40,000 or more. Household income was treated as a continuous as well as a dichotomous variable. As dichotomous measures, we categorized household income as (0) US$0–19,999 versus (1) US$20,000 or more. 

Education Attainment: Education attainment was self-reported. Levels of education attainment: (1) 11 years or less; (2) 12 years; (3) 13–15 years; (4) 16–17 years; and (5) 18+ years. Education attainment was treated as a continuous variable as well as a dichotomous variable. As a dichotomous measure, we categorized education attainment as (0) less than 16 years versus (1) 16 years or more.

Depressive Symptoms: We used the modified version of the Center for the Epidemiological Studies–Depression (CES-D) developed by Radloff in 1977 [25] to measure depressive symptoms. The measure assesses (in the past 30 days) how often a person experienced the following symptoms: trouble keeping their mind on tasks, crying spells, being unable to get going, and feeling depressed, restless, that everything was an effort, that people were unfriendly, and that people disliked them. Response items ranged from (0) rarely or none of the time to (3) most of the time, with a sum score potentially ranging from 0 to 24. The full and modified version of the CES-D are valid in African Americans [26,27,28] (Cronbach’s alpha = 0.76).

Psychological Distress: Psychological distress was measured using the K6, developed by Kessler [29]. K6 is a six-item scale that assesses nonspecific psychological symptoms including symptoms of depression and anxiety in the past month. Participants report how often they felt sad, hopeless, worthless, nervous, restless, and had no interest in things in the past 30 days. The K6 identifies persons that are high risk for mental health problems and individuals who require psychiatric and psychological treatment [30,31]. Response items were on a scale from 1 (none of the time) to 5 (all the time), which provided a sum score with a potential range from 0 to 30. Higher scores reflected more psychological distress [31] (Cronbach’s alpha = 0.84).

### 2.5. Statistical Analysis

To consider the NSAL complex sampling design, Stata 15.0 (Stata Corp., College Station, TX, USA) was used to analyze the data. We used Taylor series approximation to recalculate the complex design-based estimates of the variance and the standard errors (SEs). All inferences as well as proportions and averages reported here are weighted and reflect the NSAL’s complex design. As a result, the inferences and rates are both reprehensive and generalizable to African American adults who reside in the nation. Using Svy commands and sub-pop options, we used several survey linear regressions for multivariable analysis. At the first step, we ruled out multi-collinearity between education attainment and other SES indicators. We also tested the assumption of linear distribution of residuals (errors) in our linear regression model. In all the models that we estimated, educational attainment was the main independent variable, while depressive symptoms and psychological distress were the dependent variables. Age and other SES indicators were covariates. First, we ran linear regressions in the pooled sample. *Model 1* did not include any interaction terms. *Model 2* included an interaction term between education attainment and gender. For stratified models, we ran linear regression models in African American males and females, separately (*Models 3*–*4*). Adjusted regression coefficients (*b*), standard errors (SEs), 95% confidence intervals (CIs) for *b*, and *p*-values were reported. A *p*-value of less than 0.05 was considered significant [32].

## 3. Results

### 3.1. Descriptive Statistics

From the 3570 African American adults who participated in the NSAL, 2299 were females and 1271 were males. Table 1 describes age, SES, depressive symptoms, and psychological distress in the overall sample, as well as by gender. African American females reported higher depressive symptoms and psychological distress compared to African American males. African American males were more likely to be married than African American females (Table 1).

### 3.2. Linear Regressions (Overall Sample)

Table 2 summarizes the results of two linear regressions with perceived discrimination as the outcome, education attainment as the independent variable, and depressive symptoms and psychological distress as outcomes. *Model 1* was estimated in the pooled sample, without any interaction term. *Model 2* included the education × gender interaction term. In the pooled sample, education attainment was inversely associated with depressive symptoms and psychological distress. In the pooled sample, we found significant interactions between gender and education attainment on depressive symptoms and psychological distress, suggesting that higher education attainment is associated with a relatively greater decline in depressive symptoms and psychological distress for female as compared to male African Americans (Table 2).

### 3.3. Linear Regressions (Stratified Models)

Table 3 presents the results of gender-stratified regression models. For African American men (*Models 3*) and African American women (*Models 3*), high education attainment was correlated with fewer depressive symptoms and psychological distress, however, the magnitude of the associations were stronger for females than males (Table 3).

## 4. Discussion

In a nationally representative sample, education attainment unequally reduced depressive symptoms and psychological distress for male and female African American adults. African American males were found to be at a relative disadvantage compared to African American females in terms of gaining mental health from their education attainment. 

The finding of this study extends the MDR theory [5,6] from a literature which is almost exclusively between race and ethnic groups to examine patterns within race and ethnicity, and suggests that it is also the intersection of other social identities such as gender that determine how much health groups and individuals gain from their SES resources. Regardless of their age, whether they are children [11,33], youth [16,34,35], adults [8], or older adults [36], SES generates less improved health for non-whites as compared to whites. Among African Americans, males are at an increased disadvantage regarding gaining mental health from the very same SES resources. As mentioned before, most previous studies built on the MDR theory have focused on a comparison of race and ethnic minority groups with whites, and less information is available on differences between gender within a particular race. 

Effects of education attainment [15] and employment [37] on mortality are weaker for African Americans as compared to whites. Education attainment [4] and income [2,3,38] better reduce risk of depression for whites as compared to African Americans. Income promotes positive emotions [39] and self-rated mental health [19] of whites but not African Americans.

African American men gain fewer psychological benefits from their education attainment and income because of the pervasive racism and discrimination [3,5,17,38,40]. As the SES of African American males increases, they are subject to more contact with whites, [38,40], which in turn increases their discriminatory experiences [16,17]. High-SES African American boys attend predominantly white schools [38] and high-SES African American men work in predominantly white work places [40]. 

Discrimination, which is shown to reduce the health gain of SES [41], is more common towards male than female African Americans [17,22]. Furthermore, African American males are more prone to the effects of discrimination and environmental stressors on distress and depression [23,24,42]. 

A very recent study by Chetty et al. showed that even amongst African Americans, males are the least likely to enjoy upward social mobility out of poverty. In their study on the intersection of race and gender on intergenerational social mobility, African American men and boys were more likely to experience downward mobility compared to African American women and girls [43]. African American males find themselves in an inescapable societal circumstance, where they are more likely to decline socially and economically despite education. Even if they do maintain their SES, however, this does not protect them from depression and other psychological ills as it does other groups. The contribution of these studies questions the idea that higher education and income improves the plight of African American men in the United States.

There is a well-established literature that helps us understand the current findings. The works of Robert Staples [44,45,46], Tommy Curry [47], Daphne Watkins [48,49,50,51], and others [52,53] on African American men are some examples. Staples argued that there is a specific disadvantage of African American males that requires specific investigation [44,45,46]. Curry mentioned that being African American and male creates a unique social matrix of oppression that nullifies many of the theories we have of gender, race, and class [47]. Daphne Watkins argues that depression manifests itself through John Henryism, where African American men are high functioning and productive, yet still psychologically suffering [48,49,50,51]. Other researchers have also published on the same issues among African American men [52,53].

The results could also be explained by the Subordinate Male Target hypothesis [54,55] and Social Dominance Theory (the Intergroup Theory of Social Hierarchy and Oppression) [56], as well as Curry’s black male vulnerability thesis [47], and the work on black masculinity by Wizdom Powell [57,58] and Derek Griffith [59,60,61,62].

African American males experience more interpersonal discrimination than African American females across U.S. institutions including but not limited to the labor market, banking, the justice system, and police [16,17,63,64]. Discrimination reduces the health gains from available SES resources, particularly education attainment [41]. Due to discrimination in the labor market, the very same education attainment generates less income for African American men than other groups [39], a pattern which also explains why employment generates less physical health for African American men [37]. Despite high education, African Americans have a higher chance of staying beneath the poverty line [65]. In a 10-year longitudinal study, high levels of education attainment predicted a larger increase in income for whites but not African Americans [39]. In the same study, neither education attainment nor income generated positive affect for African Americans, yet these effects could be seen for whites [39]. In a study using data from the Health Information National Trends Survey (HINTS), income fully explained why education attainment generated less self-rated health for African Americans compared to whites [57].

Highly educated and high-income African Americans are at an increased risk of depression [3,66]. In a longitudinal study of a representative sample, African American men were at a risk for an increased risk of depressive symptoms over time [4]. African American boys from high income families were at an increased risk of depression [2], and most educated black Caribbean women were at a higher risk of suicidal ideation [20]. In another study on a representative sample of adults from Michigan, an increase in income enhanced self-rated mental health of whites but not African Americans [19].

Our results suggest that highly educated African American males are at high risk of depressive symptoms and psychological distress. Upward social mobility is more costly for African Americans than whites [41]. In a recent study, whites’ stress was a function of social mobility, however, African Americans experienced high levels of stress regardless of social mobility [67]. In another study among American men, income reduced perceived discrimination of whites but not African Americans [68]. Thus, for African Americas, successful upward social mobility (e.g., high education attainment) is not similarly rewarded in terms of life conditions, income, purchase power, and health. 

These weak effects of education attainment in reducing depressive symptoms and psychological distress for African American men are in line with previous research [1,4] and may explain why education attainment and income better reduce risk of chronic medical disease [14], obesity [11], health risk behaviors [7,8], and mortality [15] for whites as compared to African Americans, particularly males [2,3,22]. More research is needed to understand why the diminished return of SES on health is worse for males than females.

### 4.1. Implications

The results suggest that highly educated African Americans men still show depressive symptoms and psychological distress, however, high-SES female African Americans gain mental health due to education attainment. These findings suggest the need for screening, diagnosis, and treatment of mental distress and depression for highly educated African American men. Such investment is not, however, necessary for highly educated African American women, given they gain mental health protection from each additional year of schooling. This is troubling for policy makers and program planners because traditionally, high SES is assumed to signal low risk for behavioral problems [69]. In other words, although high SES generally allows a lower investment for prevention, education, and treatment of mental health problems for populations, this is not the case for African American men. Education attainment conveys less information regarding psychological distress and depressive symptoms for male than female African American adults. This is another reason that clinical and public health interventions and programs should be tailored for the intersection of race, gender, and class. Such approach may be superior to universal programs that ignore specific needs of subsections of the society [32].

### 4.2. Limitations

The current study had a few limitations. First, the cross-sectional design limits any causal inferences. While low SES impacts psychological well-being, psychopathology may also cause downward social mobility. However, the reverse causation is more relevant to SES indicators that are more subject to change later in life (such as income, marital status, and employment) compared to educational attainment, which is commonly stable in adulthood. Second, sample size was not balanced between the gender groups, with a lower number of males being a part of this study. Differential sample size results in differential statistical power, especially for gender-stratified models. This issue was not a concern in our study because association were significant in all groups. Third, the outcome of the current study was based on symptoms rather than clinical disorders diagnosed by psychiatrists, and data were collected based on a structured clinical interview. Fourth, very few confounders were controlled for in this study. Additional individual level factors as well as contextual factors may confound the association between personal SES and psychological distress [70]. Fifth, self-report measures of distress and depression may be prone to different levels of measurement bias by gender. We cannot rule out possibility that females have a higher tendency to disclose their emotional symptoms. Sixth, gender may affect access to the health care, stigma, social desirability, and several other factors that have implications regarding distress. In addition, this study did not assess the beliefs of African American men and women with regard to structural racism, oppression, and justice [71,72,73]. Last but not least, as a large proportion of African American males are institutionalized (e.g., imprisoned), selection bias may be more problematic for males than females. There is a need to replicate these findings using longitudinal design, with other SES indicators, other age groups, and using more robust outcomes such as physician diagnosis of psychiatric disorders (e.g., depression).

## 5. Conclusions

To conclude, although higher education attainment is associated with lower depressive symptoms and psychological distress overall, this protection is reduced in male as compared to female African Americans. This finding extends the MDR theory and shows that male and female African Americans differ in social patterning of psychological distress and depressive symptoms. This finding may have implications for epidemiological studies, policies, as well as clinical practice.

## Figures and Tables

**Table 1:** Descriptive statistics in the pooled sample and by gender among African American adults.

	All African Americans (*n* = 3570)		African American Women (*n* = 2299)		African American Men (*n* = 1271)	
	Mean (SE)	95% CI	Mean (SE)	95% CI	Mean (SE)	95% CI
Age (Years) *	42.01 (0.53)	40.94–43.08	42.17 (0.57)	41.01–43.33	41.80 (0.66)	40.46–43.14
Household Income *	3.63 (0.14)	3.36–3.91	3.19 (0.11)	2.96–3.42	4.20 (0.19)	3.80–4.59
Education Attainment *	12.47 (0.09)	12.30–12.64	12.47 (0.10)	12.27–12.67	12.47 (0.12)	12.24–12.71
Psychological Distress *	4.81 (0.13)	4.54–5.09	5.16 (0.14)	4.87–5.45	4.37 (0.18)	4.00–4.74
Perceived Discrimination *	4.16 (0.10)	3.94–4.37	4.45 (0.12)	4.20–4.70	3.78 (0.14)	3.49–4.06
	% (SE)	95% CI	% (SE)	95% CI	% (SE)	95% CI
Employment						
Employed	89.93 (0.01)	88.39–91.28	88.90 (0.01)	87.13–90.45	91.24 (0.01)	88.96–93.08
Unemployed	10.07 (0.01)	8.72–11.61	11.10 (0.01)	9.55–12.87	8.76 (0.01)	6.92–11.04
Marital Status *						
Unmarried	58.36 (0.01)	56.26–60.44	64.45 (0.01)	61.81–67.01	50.62 (0.02)	47.27–53.96
Married	41.64 (0.01)	39.56–43.74	35.55 (0.01)	32.99–38.19	49.38 (0.02)	46.04–52.73

CI: confidence interval; SE: standard error. * *p* < 0.05. Source: National Survey of American Life (NSAL-2003).

**Table 2:** Summary of linear regression education attainment on depressive symptoms and psychological distress in the pooled sample of African American adults.

	All African Americans (*n* = 3570)						All African Americans (*n* = 3570)						
	Psychological Distress						Depressive Symptoms						
	*b*	SE	95% CI		*z*	*p*	*b*	SE	95% CI		*Z*	*p*	
*Model 1*													
Gender (male)	−0.68	0.18	−1.05	−0.31	−3.72	0.001	−0.56	0.16	−0.89	−0.23	−3.48	0.001	
Age (years)	−0.04	0.00	−0.05	−0.03	−8.21	0.000	−0.04	0.01	−0.05	−0.03	−7.77	0.000	
Unemployed	0.83	0.32	0.17	1.49	2.56	0.015	0.79	0.31	0.16	1.41	2.56	0.015	
Married	0.14	0.17	−0.20	0.48	0.82	0.418	−0.01	0.15	−0.32	0.30	−0.05	0.961	
Household Income (US$10,000)	−0.14	0.04	−0.22	−0.05	−3.27	0.002	−0.11	0.03	−0.18	−0.04	−3.22	0.003	
Education (years)	−0.25	0.04	−0.33>	−0.17	−6.30	0.000	−0.25	0.03	−0.30	−0.19	−8.37	0.000	
Intercept	10.31	0.56	9.18	11.44	18.54>	0.000	9.50	0.46>	8.56	10.44	20.51	0.000	
*Model 2*													
Gender (male)	−2.89	0.80	−4.52	−1.25	−3.59	0.001	−2.78	0.79	−4.38	−1.17	−3.52	0.001	
Age (years)	−0.04	0.00	−0.05	−0.03	−8.22	0.000	−0.04	0.01	−0.05	−0.03	−7.79	0.000	
Unemployed	−0.33	0.05	−0.44	−0.23	−6.52	0.000	0.76	0.30	0.14	1.37	2.50	0.018	
Married	0.80	0.33	0.14	1.47	2.45	0.019	0.01	0.15	−0.29	0.32	0.09	0.927	
Household Income (US$10,000)	0.16	0.16	−0.17	0.49	0.99	0.328	−0.11	0.03	−0.18	−0.04	−3.35	0.002	
Education (years)	−0.14	0.04	−0.22	−0.06	−3.41	0.002	−0.33	0.05	−0.42	−0.23	−7.22	0.000	
Education (years) × Male	0.18	0.07	0.04	0.31	2.68	0.011	0.18	0.06	0.05	0.31	2.81	0.008	
Intercept	11.31	0.70	9.90	12.73	16.24	0.000	10.51	0.64	9.22	11.80	16.55	0.000	

CI: confidence interval; SE; standard error. Source: National Survey of American Life (NSAL-2003).

**Table 3:** Summary of linear regression on the effects of education attainment on depressive symptoms and psychological distress in African American adults by gender.

	African American Women (*n* = 2299)						African American Men (*n* = 1271)					
	Psychological Distress						Depressive Symptoms					
	*b*	SE	95% CI		*z*	*p*	*b*	SE	95% CI		*z*	*p*
*Model 3*												
Age (years)	−0.05	0.01	−0.06	−0.04	−9.11	0.000	−0.05	0.01	−0.06	−0.04	−7.86	0.000
Unemployed	0.89	0.40	0.08	1.71	2.24	0.032	1.07	0.36	0.34	1.80	2.98	0.005
Married	0.36	0.24	−0.12	0.84	1.55	0.132	0.17	0.17	−0.18	0.52	0.99	0.331
Household Income (US$10,000)	−0.22	0.05	−0.32	−0.11	−4.31	0.000	−0.18	0.05	−0.28	−0.08	−3.67	0.001
Education (years)	−0.31	0.05	−0.42	−0.19	−5.61	0.000	−0.30	0.05	−0.39	−0.20	−6.05	0.000
Intercept	11.56	0.75	10.04	13.08	15.44	0.000	10.56	0.69	9.15	11.96	15.30	0.000
*Model 4*												
Age (years)	−0.03	0.01	−0.04	−0.01	−3.29	0.002	−0.03	0.01	−0.05	−0.01	−3.77	0.001
Unemployed	0.57	0.50	−0.44	1.58	1.15	0.257	0.20	0.41	−0.63	1.03	0.49	0.625
Married	−0.11	0.25	−0.61	0.40	−0.43	0.667	−0.19	0.27	−0.73	0.36	−0.70	0.489
Household Income (US$10,000)	−0.08	0.05	−0.18	0.02	−1.64	0.110	−0.06	0.04	−0.14	0.02	−1.57	0.127
Education (years)	−0.17	0.04	−0.26	−0.08	−3.77	0.001	−0.17	0.04	−0.25	−0.08	−4.05	0.000
Intercept	7.88	0.62	6.62	9.14	12.69	0.000	7.45	0.59	6.26	8.64	12.70	0.000

CI: confidence interval; SE: standard error. Source: National Survey of American Life (NSAL-2003).
